# Encouraging Local Control With Pediatric Spatially Fractionated Radiation Therapy

**DOI:** 10.1016/j.adro.2026.102069

**Published:** 2026-06-23

**Authors:** Roman O. Kowalchuk, Safia K. Ahmed, Daiki Hara, Hok Wan Chan Tseung, Peter J. Schoettler, Wendy A. Allen-Rhoades, Chelsea M. Self, Dawn Owen, Michael P. Grams, Anita Mahajan, Nadia N. Laack

**Affiliations:** a Department of Radiation Oncology, Mayo Clinic Rochester, Rochester, Minnesota; b Department of Radiation Oncology, Mayo Clinic Arizona, Phoenix, Arizona; c Department of Pediatrics, Division of Pediatric Hematology/Oncology, Mayo Clinic Rochester, Rochester, Minnesota; d Department of Pediatric Hematology Oncology, Children’s Minnesota, Minneapolis, Minnesota; e Johns Hopkins Pediatric Radiation Oncology Research Center at Sibley Memorial Hospital, Washington, District of Columbia

## Abstract

**Purpose:**

Spatially fractionated radiation therapy (SFRT) is a promising treatment involving the delivery of discrete spheres of high-dose radiation, potentially enhancing treatment response. Despite encouraging results for adult patients, no SFRT data exist in pediatric patients.

**Methods and Materials:**

All pediatric patients receiving radiation therapy from 2019 to 2025 with at least 1 follow-up imaging study were considered. Patient characteristics and outcomes were recorded via institutional review board–approved retrospective chart review.

**Results:**

Five pediatric patients who received SFRT were identified, with a median age of 15.7 years (range, 6.5-17.2 years). Three were alive at last follow-up (median, 13 months; range, 3-18 years). Neither tumor progression nor grade ≥ 2 toxicity was identified at SFRT sites. One recurrent wound infection occurred after enoxaparin sodium injection in the high-dose radiation therapy field. Two patients received upfront SFRT for pelvic osteosarcoma, followed by 74.8 Gy in 34 fractions and 69 Gy in 30 fractions. The treatment volume was over 600 cm^3^ in both cases, allowing for delivery of 26 and 46 high-dose spheres. Both patients remain alive with primary tumor control; however, both have metastatic disease. Three other cases treated metastatic and/or recurrent disease: Ewing sarcoma, anaplastic ependymoma, and renal cell carcinoma. Sites included chest wall (n = 2), lung (n = 5), and pelvis (n = 1). One patient had 4 courses of SFRT, including 3 small lung volumes < 20 cm^3^ allowing delivery of 2 to 5 spheres per treatment.

**Conclusions:**

We present one of the first case series of SFRT in pediatric patients, with favorable local control and toxicity results. Further study of SFRT for radioresistant or recurrent tumors is required.

## Introduction

Spatially fractionated radiation therapy (SFRT) is a promising treatment involving the delivery of discrete spheres of high-dose radiation, potentially enhancing treatment response.^1^ The emergence of this modality represents the continued development of radiotherapeutic strategies to deliver high doses of radiation therapy to increasingly conformal treatment volumes while maximizing local control and minimizing toxicity, as shown with stereotactic body radiation therapy (SBRT) across multiple primary and metastatic settings.^2^, ^3^, ^4^ This includes treatment of pediatric Ewing sarcoma and osteosarcoma, tumors with intrinsic radioresistance which can reduce local control.^5^^,^^6^ With conventional fractionation, treatment of osteosarcoma requires doses over 70 Gy to maximize local disease control, and even when such doses are achieved, local failure remains a significant factor in overall disease progression.^7^

A phase 1 trial studying lattice SBRT for large tumors found minimal toxicity associated with lattice SBRT.^8^ Building on these results, our group has published extensively on optimizing SFRT planning approaches: lattice, brass, volumetric modulated arc therapy, and protons.^9^, ^10^, ^11^, ^12^ We have increasingly offered SFRT for adult patients with large tumors, particularly sarcomas. A recent publication regarding of 176 patients revealed 81% 1-year local control and grade 3 toxicity in only 9 patients.^13^ Study of patients with sarcoma revealed similar findings: 82% 1-year local control, 60% symptom relief, and 8% grade ≥ 3 toxicity.^14^

Despite encouraging results for adult patients, no SFRT data have been reported in pediatric populations. Therefore, we present one of the first series of pediatric patients treated with SFRT, with the goal of demonstrating an early signal of treatment efficacy and minimal treatment-related toxicity.

## Methods

### Cohort development

All pediatric patients receiving radiation therapy from 2019 to 2025 with at least 1 follow-up imaging study were considered. Patient characteristics and outcomes were recorded via institutional review board–approved retrospective chart review. The heterogeneity of this cohort reflects the diverse clinical indications that prompted consideration for SFRT. Though each case represents a nuanced clinical scenario, SFRT was chosen in 2 cases of osteosarcoma because of poor long-term local control with definitive radiation therapy alone and 3 cases involving both radioresistant, recurrent tumors overlapping with prior radiation therapy. The study was conducted in accordance with the Declaration of Helsinki and approved by the Institutional Review Board at Mayo Clinic.

### SFRT planning

SFRT was delivered using proton-based lattice therapy or photon-based mini-lattice therapy. Both SFRT approaches were prescribed a dose of 20 Gy to high-dose spheres. Proton spot-scanning based lattice plans used a minimum of 2 fields with opening angles > 40° delivering dose to 0.8- to 1-cm diameter spherical targets placed >20 mm apart within the gross target volume. The plans were normalized such that 50% of the total sphere volume received the prescription dose. Photon-based mini-lattice plans used 1 or 2 partial arcs with a 6-MV flattening filter free beam. Four-mm spherical mini-lattices were placed 1 cm apart in the cranial-caudal axis throughout the gross target volume and positioned at least 1 cm away from any organs at risk (OARs). Mini-lattice plans used dynamic conformal arcs to conform multi-leaf collimators to the spherical mini-lattices and to separate each opening with a closed multi-leaf collimator leaf pair. Mini-lattice plans were normalized such that each mini-lattice volume received a minimum of the prescription dose.

### Statistical analysis and outcomes assessment

Given the small number of patients for whom SFRT was delivered, descriptive statistics and outcomes were used. Follow-up duration and overall survival were calculated from the first date of SFRT, thereby accounting for different follow-up external beam radiotherapy (EBRT) regimens. Grade ≥ 2 (G2+) toxicity at least possibly related to SFRT was recorded using retrospective chart review.

## Results

**Figure 1:**
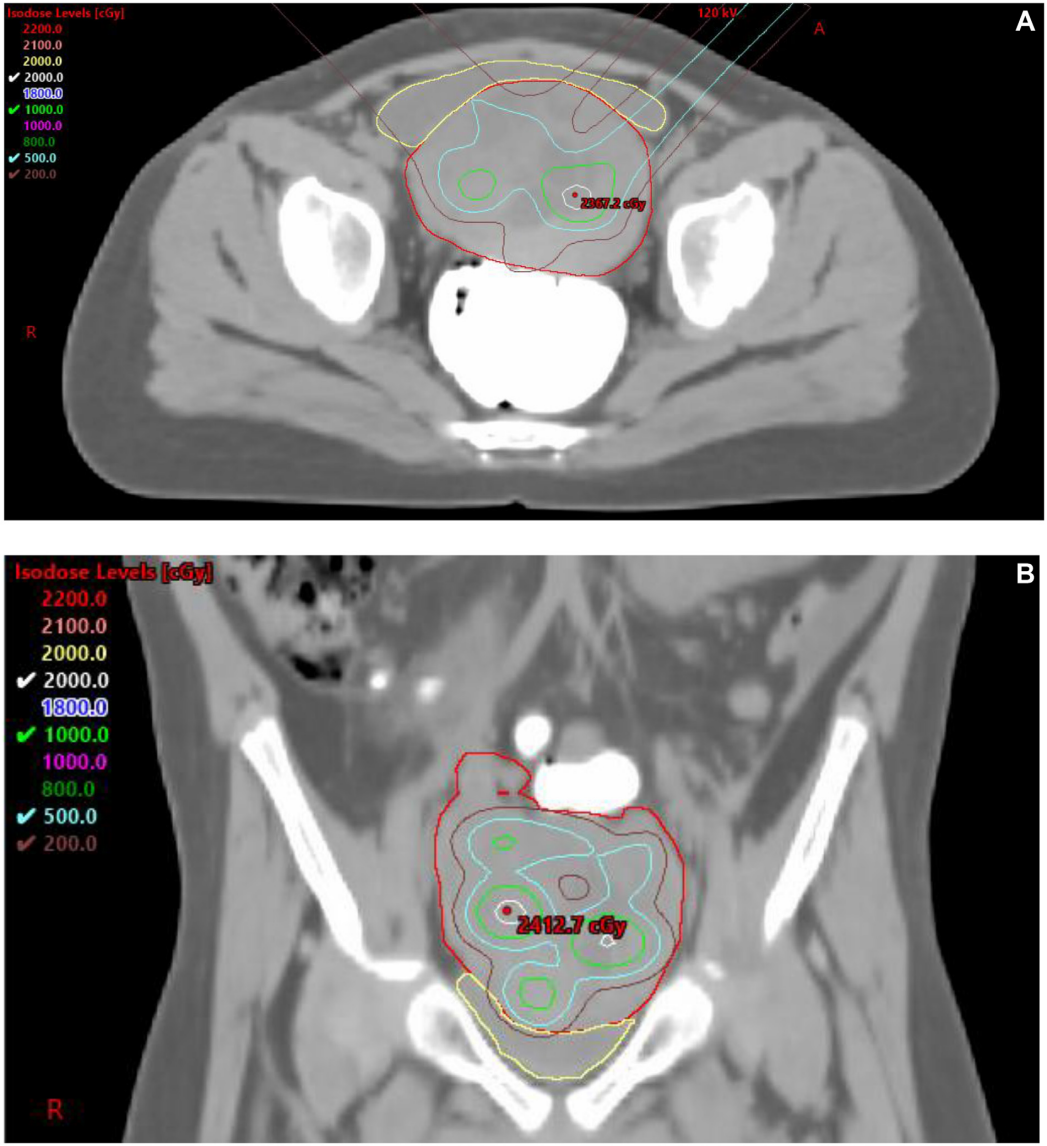
Spatially fractionated radiation therapy was used to treat pelvic anaplastic ependymoma. Treatment delivery reveals high-dose spheres of ≥20 Gy with only minimal dose exposure to organs at risk (eg, bladder, yellow). Representative (a) axial and (b) coronal slices are shown.

Five pediatric patients who received SFRT were identified, with a median age of 15.7 years (range, 6.5-17.2 years) (Table 1). Three were alive at last follow-up (median, 13 months; range, 3-18 years). Only 1 patient died within 1 year of SFRT (Table 2). All other patients had at least 8 months of clinical and imaging follow-up. Two patients had prior radiation therapy with overlap of the SFRT target; 2 patients had no prior radiation therapy; and 1 patient had prior radiation therapy with no overlap with the SFRT target. Ten total SFRT treatments were delivered. Because 3 planned treatments were delivered to a single target, 8 SFRT target sites were treated. Eight of 10 treatments involved proton-based SFRT, and 2 used photon-based planning. Most cases involved follow-up EBRT (Fig. 1). Follow-up EBRT was only omitted in 2 cases with overlap of prior radiation therapy. Follow-up regimens varied, but hypofractionated radiation therapy or SBRT were often delivered (n = 4).

**Table 1:** A brief description of the 5 treated patients is provided

Patient	Age at SFRT start (y)	Primary tumor type	Treatment situation	Prior radiation therapy	SFRT site(s)	SFRT site(s) volume (cm^3^)
1	10.7	Ewing sarcoma	Recurrence	Yes, overlapping	Chest wall, lung	38.4, 13.4, 15.8, 16.1
2	6.5	Anaplastic ependymoma	Recurrence	Yes, no overlap	Pelvis	107.5
3	15.7	Osteosarcoma	Upfront	No	Pelvis	638.6
4	16.4	Osteosarcoma	Upfront	No	Pelvis	927
5	17.2	Renal cell carcinoma	Recurrence	Yes, overlapping	Lung, chest wall	360.8, 112.1, 67.2

*Abbreviation:* SFRT = spatially fractionated radiation therapy.

**Table 2:** A description of treatment outcomes for the 5 treated patients is provided

Patient	Radiation therapy dose / fraction (Gy)	G2+ toxicity	Alive at last follow-up	Clinical follow-up duration (mo)	Notes
1	20/1->40/10; 20/1; 20/1; 20/1	None	No	13.5	One site (lung) treated with 3 planned SFRT treatments
2	20/1->40/5	None	No	3.0	
3	20/1->74.8/34	None	Yes	8.7	Upfront osteosarcoma treatment for local control
4	20/1->69/30	None	Yes	15.1	Upfront osteosarcoma treatment for local control; Recurrent wound infection after enoxaparin sodium injection in site of high-dose RT
5	20/1->30/5; 20/1->50/5; 20/1	None	Yes	18.0	

*Abbreviations:* G2+ = grade ≥ 2; RT = radiation therapy; SFRT = spatially fractionated radiation therapy.

**Figure 2:**
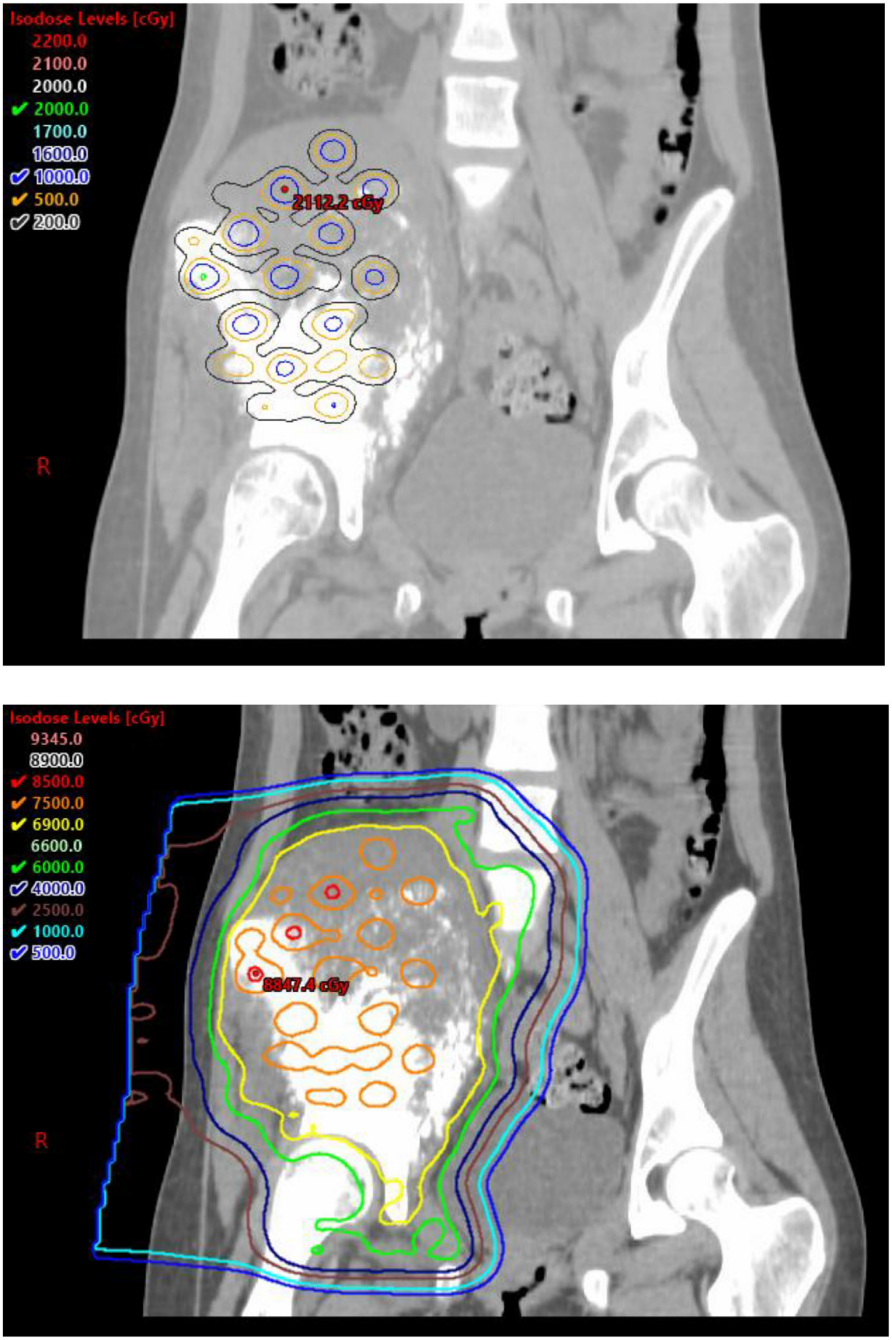
A case example of spatially fractionated radiation therapy (SFRT) use in the upfront treatment of pelvic osteosarcoma is shown. Representative coronal slices of the SFRT plan (top) and sum plan after subsequent delivery of 69 Gy in 30 fractions (bottom) are shown. The primary tumor decreased in size after completion of radiation therapy, and no subsequent primary tumor progression was identified.

Two patients received SFRT for pelvic osteosarcoma at the time of definitive radiation therapy for local control. In both cases, surgical resection was deemed either not feasible and/or associated with unacceptable morbidity for the patient. SFRT was followed by 74.8 Gy in 34 fractions and 69 Gy in 30 fractions (Fig. 2). The treatment volume was over 600 cm^3^ in both cases, allowing for delivery of 26 and 46 high-dose spheres. Both patients remain alive with primary tumor control; however, both have metastatic disease. Such a treatment strategy represents the first known application of SFRT in the upfront treatment paradigm for pediatric patients.

**Figure 3:**
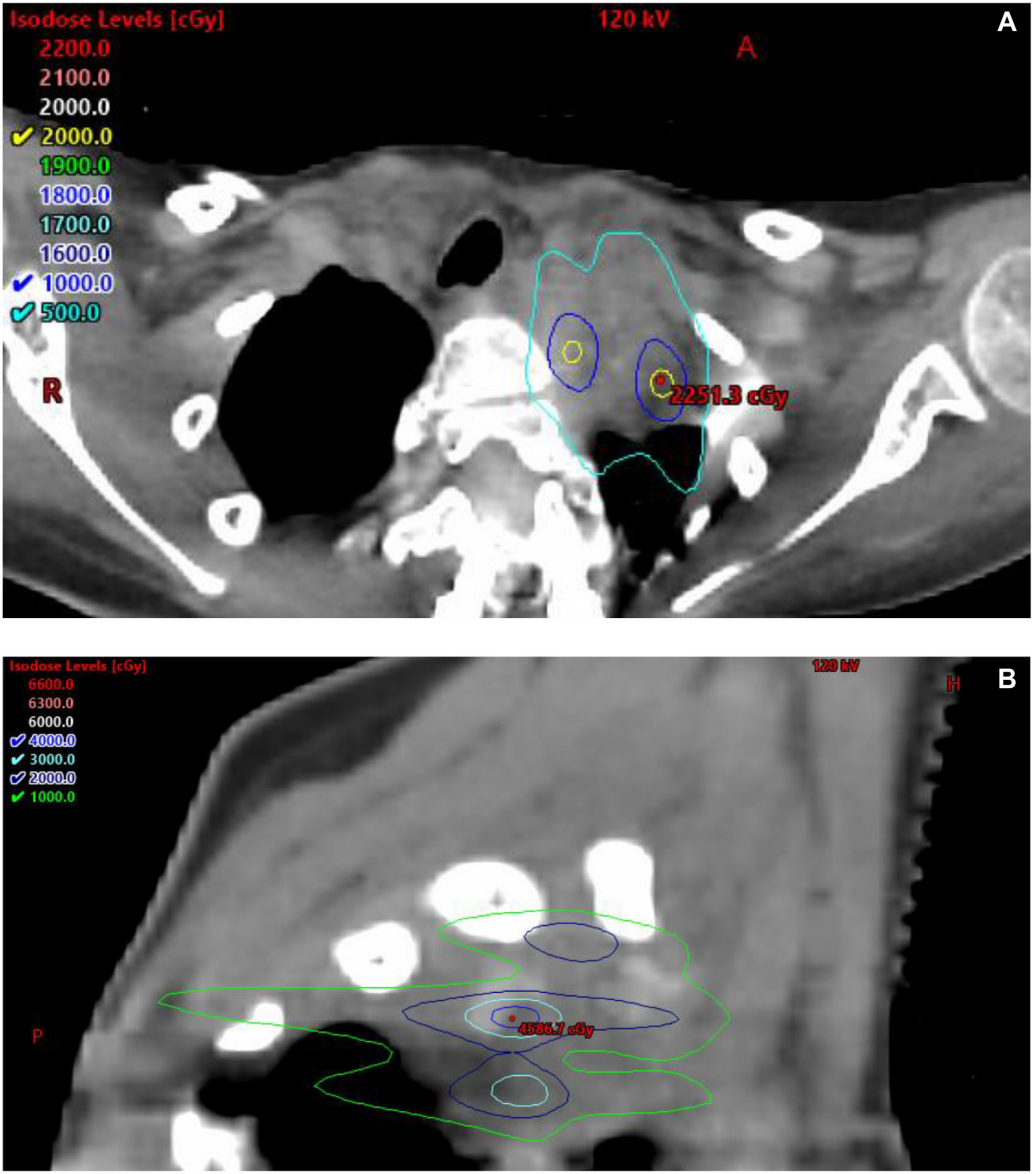
A case example of serial spatially fractionated radiation therapy (SFRT) use for a lung metastasis in the setting of prior radiation therapy is demonstrated. (a) A characteristic slice from one of the serial plans is shown, as well as (b) a sagittal slice from the sum plan. Unfortunately, the patient passed away 3 months after completing the first SFRT fraction.

Overall, treatment volume and delivered SFRT spheres varied dramatically (median, 87.4 cm^3^, 4.5 delivered spheres). Three other cases used SFRT to treat metastatic and/or recurrent disease: Ewing sarcoma, anaplastic ependymoma, and renal cell carcinoma. Sites included chest wall (n = 2), lung (n = 5), and pelvis (n = 1). One patient had 4 courses of SFRT, including 3 small lung volumes < 20 cm^3^ allowing delivery of 2 to 5 spheres per treatment (Fig. 3). The latter treatment approach of 3 SFRT treatments within approximately 2 months was, to our knowledge, a novel application of SFRT. This treatment involved 3 planned SFRT courses to metastatic disease for recurrent Ewing sarcoma. No follow-up EBRT was delivered after these treatments, and the SFRT spheres were intentionally positioned in different tumor regions with each treatment, such that a larger tumor volume would be treated with the high-dose spheres. This approach stabilized the treated the lesion despite initial radioresistance to upfront radiation therapy; however, the patient unfortunately died.

**Figure 4:**
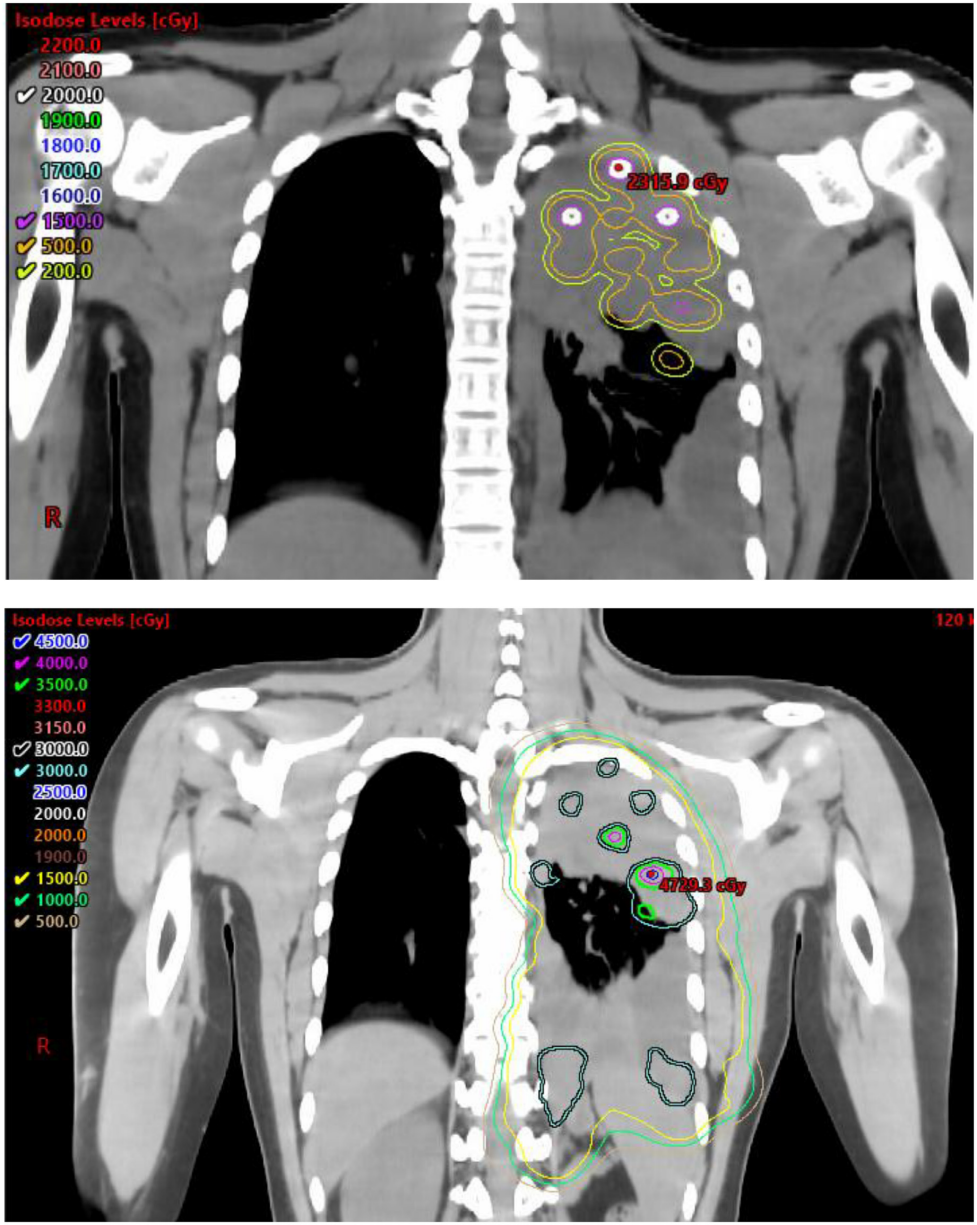
Spatially fractionated radiation therapy (SFRT) was delivered for a lung metastasis from primary renal cell carcinoma (Patient 5). Representative slices of the SFRT plan (top), as well as the sum plan after subsequent delivery of 30 Gy in 5 fractions (bottom) are shown. This patient had prior radiation therapy with overlap of this treated site, leading the treating physician to offer a lower subsequent dose of radiation therapy, compared with more conventional SBRT doses.

Another patient had 3 sites treated with SFRT (Fig. 4). The first was for a lung site, and the latter 2 involved chest wall and lung sites. In the first 2 treatments, SFRT was followed by the delivery of 30 Gy in 5 fractions and 50 Gy in 5 fractions. No follow-up SFRT was delivered for the third treatment.

No tumor progression was identified at last follow-up for any SFRT sites. Of the 8 total treated sites, 3 were stable, and 5 decreased in size. Applying Response Evaluation Criteria in Solid Tumors (RECIST) demonstrated that 3 target sites had partial response, and all others showed stable disease.

No G2+ toxicity was identified at SFRT sites. One recurrent wound infection occurred after enoxaparin sodium injection in the high-dose radiation therapy field. It is unclear whether this was directly a result of the SFRT, the follow-up EBRT, and/or suboptimal injection site selection.

Systemic therapy use was variable across the described cases. Four of 7 cases involved concurrent systemic therapy (ranging from cisplatin, doxorubicin, and methotrexate; bevacizumab and erlotinib; and vincristine, irinotecan, and temozolomide). The eighth case (with the 3 SFRT treatments for the small lung volume for recurrent Ewing sarcoma) had concurrent pembrolizumab for 2 of 3 fractions. There was similar heterogeneity of systemic therapy after SFRT, ranging from bevacizumab and erlotinib to continuing vincristine, irinotecan, and temozolomide.

Overall outcomes for this cohort were poor. All patients experienced subsequent out of field recurrence. For 4 of 8 targets, this occurred in <4 months. Three cases involved recurrence between 6 and 9 months after SFRT, and the final case involved recurrence 14 months after SFRT.

## Discussion

We present one of the first case series of SFRT delivery in pediatric patients. Across a heterogeneous set of clinical `scenarios, incorporation of SFRT within the treatment paradigm resulted in target site control in all cases without any documented G2+ toxicity secondary to SFRT.

Relapsed pediatric sarcomas are associated with high rates of disease progression, and clinical outcomes have shown little improvement in recent years. For example, cabozantinib—currently under investigation by the Children’s Oncology Group—was considered promising in a phase 2 study only if at least 5 of 20 patients achieved an objective response.^15^ These sobering results highlight the urgent need for strategies that enhance both systemic and local control. Osteosarcoma, in particular, is highly radioresistant; in this report, we describe 2 cases in which SFRT was used to intensify radiation therapy dose delivery. Both patients achieved local control without any grade ≥2 toxicity. These patients also did not have to undergo surgical resection, which would have been associated with substantial morbidity. Finally, patients were able to maintain systemic therapy without meaningful delays, in contrast to significant delays required for surgical resection.

Even in cases of overlap with prior courses of radiation therapy, SFRT delivers very minimal doses to OARs, thereby offering the potential to even improve on the safety profile of SBRT. Though SBRT delivers a heterogeneous dose to the tumor to facilitate sharp dose falloff and reduction in OAR doses, SBRT plans intentionally treat the entire target volume to a given prescription dose. SFRT follows an inherently different treatment paradigm in that substantial portions of the target are spared from radiation therapy. As shown in this case series, SFRT delivery was feasible even in cases with overlapping prior radiation therapy. Therefore, SFRT could be applied in a range of clinical settings, including for metastatic disease as well as primary tumor relapse.

SFRT presents substantial translational promise which may improve local and distant tumor control. SFRT may induce transient vascular permeability, potentially allowing for improved systemic therapy response of the primary tumor.^16^ Extrapolating from SBRT data, SFRT could similarly impact the tumor microenvironment, stimulate systemic immune response, and synergize with checkpoint inhibition.^17^^,^^18^ This translational underpinning supports the oncologic thinking used for case 1, in which “pulsed” SFRT was used without follow-up radiation therapy treating the entire tumor volume.^19^ We strongly support additional translational work in this space to facilitate hypothesis-driven clinical trial design.

We acknowledge that despite this significant potential to improve outcomes, this initial report is hypothesis-generating and does not provide a direct comparison to either standard palliative regimens or SBRT. Despite encouraging outcomes for SBRT in the metastatic setting, multiple of these cases had tumor volumes too large for conventional SBRT regimens; therefore, they were not selected. Additionally, despite the impressive normal tissue sparing with SBRT, the dosimetric advantages with SFRT are even more substantial, offering the potential to reduce to healthy tissues in the setting of reirradiation and thereby potentially offering an even safer treatment regimen than SBRT. Despite overall favorable local control with radiation therapy for conventionally fractionated, palliative radiation therapy regimens, long-term local control for radioresistant, treatment refractory lesions may be as low as 10%, with fewer than 30% of patients achieving improved motor function.^20^

## Conclusions

We present one of the first case series of SFRT in pediatric patients, with encouraging local control and toxicity results. Further study of SFRT for radioresistant or recurrent tumors is required, as these preliminary data suggest the potential for SFRT to meaningfully improve patient outcomes without significantly increasing toxicity.
